# Predicting later ADHD presentation types from early childhood autism and intellectual disability

**DOI:** 10.1007/s00787-025-02805-7

**Published:** 2025-07-17

**Authors:** Yunyi Ren, Agnieszka Mlodnicka, Catrina Andaya Calub, Irva Hertz-Picciotto, Julie B. Schweitzer

**Affiliations:** 1 Department of Public Health Sciences, University of California, Davis, Davis, CA USA; 2 Department of Psychiatry and Behavioral Sciences, University of California, Davis, Sacramento, CA USA; 3 MIND Institute, University of California, Davis, 2825 50th St, Sacramento, CA USA

**Keywords:** Autism, ASD, ADHD, DD, ADHD presentation, ADHD subtypes, Developmental trajectory

## Abstract

**Background:**

Attention deficit/hyperactivity disorder (ADHD) and autism are both neurodevelopmental disorders associated with functional impairment in social, academic, and occupational domains.

**Methods:**

This longitudinal study, a follow-up to the CHARGE Study (ReCHARGE), evaluated diagnosis of ADHD and its presentation type (Inattentive, Combined-Hyperactive/Impulsive), in a cohort of 8 to 20 year-olds from four developmental categories recruited at ages 2–5 years: Autism, developmental delay without autism (DD), other early concerns (OEC) or typical development (TD, controls from the general population) (*n* = 645). ADHD diagnosis was based on parent clinical interviews (DISC or MINI), observational methods and multiple rating scales. Multivariate Poisson log regression models were fit to estimate associations of early childhood neurodevelopment with later ADHD diagnoses. Adjusted confounding variables included child’s age, sex, parental ADHD, socioeconomic indicators, and maternal prenatal conditions.

**Results:**

Of 645 participants, 213 (33.0%) met criteria for ADHD. Early childhood diagnosis was the strongest predictor. For Hyperactive/Impulsive or Combined presentation, relative risks (RRs [95% CI]) were 5.4 [3.0, 9.4] for autism, 4.4 [2.3, 8.4] for DD, and 3.1 [1.5, 6.1] for OEC. For Inattentive presentation, RRs were 2.6 [1.6, 4.2] for autism, 1.4 [0.7, 2.9] for DD, and 2.6 [1.4, 4.2] for OEC. For any ADHD presentation, RRs were 3.1 [2.2, 4.4] for autism, 2.4 [1.6, 3.6] for DD, and 2.4 [1.6, 3.6] for OEC.

**Conclusions:**

This study reinforces the need for evaluation of ADHD and its presentation type in autistic children and other developmental delays, as these youth are at high risk for ADHD. Clinicians should assess the presence of ADHD-related challenges across development and service needs in individuals with autism and/or DD due to their high ADHD risk.

**Supplementary Information:**

The online version contains supplementary material available at 10.1007/s00787-025-02805-7.

## Background

Attention deficit/hyperactivity disorder (ADHD) and autism are both neurodevelopmental disorders associated with functional impairments in social, academic, and occupational domains [1]. There are three presentations, or subtypes, of ADHD: Predominantly Inattentive, Predominantly Combined, and Predominantly Hyperactive/Impulsive (Hy/Imp) types [1]. However, the distinctiveness of the Hy/Imp presentation tends to diminish beyond early childhood. As children mature, hyperactive and impulsive symptoms become less prominent, while inattention symptoms become more apparent, particularly in school settings. As a result, many children who initially meet criteria for the Hy/Imp later qualify for the Combined presentation, which includes both inattentive and hyperactive/impulsive symptoms [2]. Autism is characterized by impairments in social communication and interaction along with restricted, repetitive behaviors or interests [1]. Given that a high proportion (40–70%) of autistic children exhibit clinically elevated ADHD symptoms [3, 4] and that early identification and treatment have been shown to be beneficial in improving ADHD symptoms and ameliorating adverse functional outcomes [5, 6], understanding early developmental predictors of ADHD and specific presentations in youth may prove critical.

Prior to the publication of DSM-5 in 2013, the presence of an autistic diagnosis excluded a diagnosis of ADHD. Due to this relatively recent introduction of the possible co-morbidity of autism and ADHD, a limited number of longitudinal studies have investigated predictors of ADHD in autistic youth. One study that used group-based trajectory modeling among autistic individuals found that higher adaptive skills in early childhood were significantly associated with lower odds of belonging to a class characterized by clinically elevated ADHD symptoms in late childhood [7]. Another study found an ADHD diagnosis during middle childhood predicted adolescent ADHD in autistic individuals, but this relationship lost significance when considering study design and attrition [8].

Few studies have assessed ADHD symptoms in early childhood, when symptoms may first emerge in autistic children [3, 9, 10]. An analysis from the CHARGE study [3] addressed this gap using a large cohort of children aged 2–5 years. ADHD symptoms were most prevalent in the autistic group, followed by those with developmental delays without autism (DD), and least common in typically developing (TD) children (1%). Autistic preschoolers with ADHD symptoms also showed greater cognitive and behavioral impairments. As the children were young, the focus was on symptoms rather than diagnosis.

The current ReCHARGE study (Revisiting CHARGE) extends the CHARGE study by examining ADHD diagnoses at ages 8–20 in children initially enrolled at ages 2–5, building on the prior work of Lyall et al. (2017) on ADHD symptoms [3], now that ADHD diagnoses may be more valid and reliable. We aimed to identify early predictors of ADHD diagnoses across groups classified in early childhood as: autistic, DD, Other Early Concerns (OEC), or TD. We hypothesized that an early autism diagnosis would predict higher rates of later ADHD, particularly Combined and Inattentive presentations, with Hy/Imp being the least common [2]. We also examined predictors of ADHD subtypes, considering sociodemographic factors, maternal prenatal conditions, birth variables, and parental histories of ADHD. The primary study rationale is that understanding early childhood precursors of ADHD can lay the foundation for early screening of ADHD and its subtypes among those with known higher risks.

## Methods

### Participants

Early childhood diagnoses in CHARGE were based on the Autism Diagnostic Observation Schedule (ADOS) [11] and the Autism Diagnostic Interview-Revised (ADI-R) [12] to confirm the autism diagnosis. Cognitive and adaptive functioning were assessed using the Mullen Scales of Early Learning (MSEL) [13], and the Vineland Adaptive Behavior Scales, Second Edition (VABS-II). Children without autism were classified as: DD (without autism); TD (recruited from general population and with no cognitive or adaptive impairments and screened negative for ASD). A fourth group with an exploratory analysis consisted of children with OEC because of a community diagnosis of autism or DD or a designation of high risk prior to participation in the CHARGE study, but who did not meet criteria based on clinician and gold standard autism assessments, nor DD or TD in the CHARGE study [14].

For this study 985 CHARGE participants also enrolled in ReCHARGE during mid-childhood or adolescence and met inclusion criteria: (1) completed early childhood study assessment with one of four exit diagnoses, (2) aged 8–20 years, and (3) resided within 3–4 h of our assessment site. Written informed consent was obtained from parents of minors and adult participants without verbal capacity, with assent from verbal children (ages 8–17) and written consent from verbal adults (ages 18–20). The project was approved by the UC Davis Institutional Review Board.

Among the 985 participants, we excluded those missing the ADHD interview (*N* = 260), and those with intellectual and adaptive impairments (Total *N* = 80 excluded; ASD *N* = 43; DD *N* = 32; OEC *N* = 3; TD *N* = 2). Intellectual impairments were determined based on the Stanford Binet Intelligence Scale if the mental age is less than 6 years (pre-COVID; *N* = 29) and the adaptive behaviors impairments on the Parent-reported Adaptive Behavior and Clinician Review after clinician’s evaluation (post-COVID; *N* = 51). Valid interviews were obtained for the remaining 645 participants in this analysis.

### Assessing ADHD diagnostic outcomes at time 2

In ReCHARGE, ADHD diagnoses at Time 2 were based on *Diagnostic and Statistical Manual of Mental Disorders*, Fourth Edition, Text Revision (DSM-IV-TR) and later Fifth Edition (DSM-5) criteria [1, 15]. Ph.D.-level psychologists integrated clinical interviews, rating scales, and behavioral observations to make diagnostic determinations. Beginning in 2017, parent interviews used the Diagnostic Interview Schedule for Children (DISC) [16]; subsequently, starting in August 2018, the updated Mini-International Neuropsychiatric Interview for Children and Adolescents (MINI-KID version 7.0.2) was used. For participants over 18 years, the MINI for adults was administered using parent report [17]. Both interviews assessed the nine ADHD Hyperactive/Impulsive and nine ADHD Inattentive symptoms, requiring more than six symptoms for those under 16 years or more than five for those over 17, plus age of onset and impairment. Clinicians reviewed all data and completed confirmation forms, noting agreement or disagreement with algorithm-based diagnoses. Discrepancies were resolved by PhD-level diagnosticians.

Additional measures informing ADHD diagnoses included the clinician-rated Behavioral Rating Inventory for Children (BRIC) [18]; parent-rated Conners’ Rating Scale – 3 (CPRS) [19]; and the parent-rated Conners’ Adult ADHD Rating Scale (CAARS) Observer Form – Long Version on the young adults [20]. T-scores > 65 on DSM ADHD scales of the CPRS or CAARS indicated ADHD endorsement. In complex cases and for the assessment of other or co-occurring psychiatric disorders, additional routinely collected measures were reviewed, including the Time 2 Aberrant Behavior Checklist (ABC), the Child Behavior Checklist (CBCL), the Barkley Adult ADHD Rating Scale (for young adults), and for those with intellectual impairments, the Scale of Attention in Intellectual Disability (SAID) [21]. Final diagnoses were made by PhD-level clinical psychologists integrating all clinical and contextual data—particularly when standard ADHD items were not well-suited (e.g., for individuals with significant intellectual delays). Psychiatric comorbidities were assessed beyond the clinical interviews with supplementation by the ECHO PROMIS mood and anxiety scales, completed by parents and cognitively able participants. Learning disabilities were identified via parent report. Table 1 lists all ADHD-related instruments used across CHARGE and ReCHARGE.

**Table 1:** Instruments table of analytical diagnoses variable used at each study time point from CHARGE to recharge

Time Point	Study	Age Range	Analyzing Diagnoses	Instruments
T1	CHARGE	2–5 Years	Autism Developmental Delay without Autism (DD) Other Early Concerns (OEC) Typical Development (TD)	Aberrant Behavior Checklist (ABC) Autism Diagnostic Observation Schedule (ADOS) Autism Diagnostic Interview-Revised (ADI-R) Mullen Scales of Early Learning (MSEL) Vineland Adaptive Behavior Scales, Second Edition (VABS-II)
T2	ReCHARGE	8–20 Years	Attention Deficit/Hyperactivity Disorder (ADHD)	MINI-Kid/MINI-Adults/DISC* Aberrant Behavior Checklist (ABC) ASEBA Child Behavior Checklist (CBCL) Behavioral Rating Inventory for Children (BRIC) Conners’ Adult ADHD Rating Scale (CAARS) Parent Conners’ Rating Scale – 3 (CPRS) Patient-Reported Outcomes Measurement Information System (PROMIS) Scale of Attention in Intellectual Disability (SAID)

* The Mini-International Neuropsychiatric Interview (MINI) or the Diagnostic Interview Schedule for Children (DICS) was used depending on the participant’s age (adult vs. child) and the timing of evaluation (before or after August 2018)

### Statistical analysis

Basic demographic information was analyzed across No-ADHD, any (Total) ADHD, and the three ADHD presentation types (Inattentive, Hy/Imp, and Combined). Bivariate comparisons were conducted using chi-square tests, ANOVA, or linear regression, depending on the variable type.

Separate sets of multivariate Poisson regression analyses were conducted for each of the three ADHD outcomes: Combined or Hyperactive/Impulsive (Combined-Hy/Imp) presentation, ADHD Inattentive presentation, and Total ADHD presentation. Each set of regression analyses followed the same procedures. All models include the CHARGE early childhood developmental diagnosis (Autism, DD, OEC, TD), as the primary predictor variable, with TD as the reference. In each of the four models, a binary ADHD outcome was compared with the no ADHD group. Predictive variables considered as potential confounders include child sex, preterm status (gestational age < 37 weeks), T-score of birthweight for gestational age, child race/ethnicity, regional area of childbirth, parents’ education, health insurance status at delivery, maternal age, maternal smoking during pregnancy, and mother’s metabolic condition, age the MINI/DISC was administered and the parents’ self-reported ADHD history. Multiple imputation was used to robustly assign values to missing predictor variables for the multivariate analysis, and variables with more than 20% missing values were neither imputed nor included in the multivariate analysis (see Appendix 1).

In our multivariate models, we excluded certain strong early childhood predictors (2–5 years) to avoid redundancy. CHARGE Mullen was omitted as it was part of diagnostic group classification. The CHARGE ABC was excluded due to overlap with ADHD symptom domains (e.g., hyperactivity, inattention) which dominated prediction of adolescent ADHD, limiting valid estimation of independent effects. ReCHARGE Conners’ scores were also excluded as they were used directly in ADHD diagnosis.

The multivariate model variable selection process involved a combination of preliminary covariate screening and an assessment of change-in-estimates for the primary exposure. First, we identified a set of theoretically relevant potential confounders (listed above) and conducted univariate Poisson regressions [22], retaining variables with a p-value less than 0.2 for further consideration. Next, a directed acyclic graph (DAG, Appendix 3) was employed to scrutinize potential confounding factors identified in previous work on early childhood neurodevelopmental conditions and in bivariate association analyses with ADHD, taking account plausibility of causal associations and avoidance of redundancy. In the multivariate Poisson regression analysis, variables were further evaluated using a change-in-estimates approach, where a change > 10% in the estimated effect of autism, DD, or OEC was used to define a confounder. The likelihood ratio test (*p* < 0.2) was also used to retain variables that may not be confounders but contributed substantively to outcome prediction. As a result, the final models may differ slightly across ADHD outcomes. Interaction terms were also considered and included where theoretically justified and supported by exploratory analysis. Results from the univariate and final multivariate Poisson-log regression models are summarized in Appendix Tables 4 and 5. To ensure no potential multicollinearity among predictors, we also examined variance inflation factors. For each predictor, we reported the estimated relative risks, the 95% confidence interval based on 20 multiple imputations, and the corresponding p-value. All analyses were performed using SAS Studio 3.8 Enterprise Edition [23].

### Model evaluation

Our primary objective is to estimate the impact of our variable of interest—the early childhood Diagnostic Group—on middle childhood or adolescent ADHD diagnosis, while accounting for sociodemographic, maternal prenatal conditions, and birth variables, along with parental histories of ADHD. The efficacy of the multivariate prediction model was assessed using the area under the receiver operating characteristic curve (AUC). Calibration plots were generated to evaluate the alignment between observed values and the predicted probabilities of ADHD diagnoses as determined by the current evaluation.

## Results

**Fig. 1:**
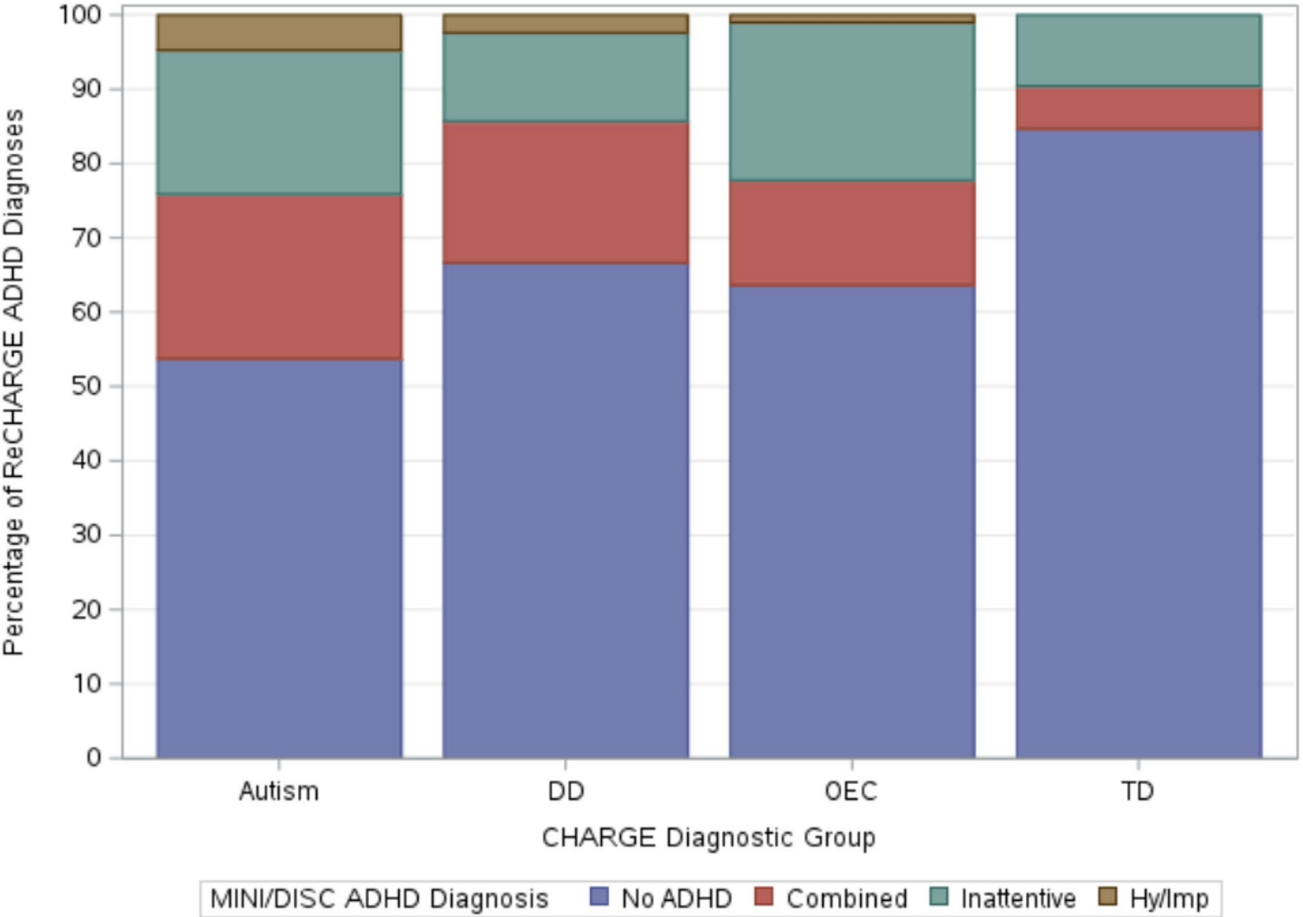
ADHD diagnoses by diagnostic group; figure displays the percentage of each ADHD diagnosis in CHARGE diagnostic group DD = developmentally delayed; OEC = other early concern; TD = typically developing

Out of the 645 participants analyzed for this study, 213 (33.0%) met criteria for ADHD: 98 (15.2%) for the Combined presentation, 100 (15.5%) for the Inattentive presentation, and 15 (2.3%) for the Hy/Imp presentation. The remaining 432 participants (67.0%) did not meet criteria for ADHD. When stratified by early childhood diagnostic group, the overall prevalence of ADHD was highest in the autism group (117 of 253; 46.2%), followed by the OEC group (36 of 99; 36.4%), the DD group (28 of 84; 33.3%), and the TD group (32 of 209; 15.3%). The autism group also showed the highest prevalence of the Combined (*n* = 56; 22.1%) and Hy/Imp presentations (*n* = 12; 4.7%) compared to other groups, whereas the OEC group had the highest prevalence of the Inattentive presentation (*n* = 21; 21.2%). Within the TD group, ADHD prevalence was lowest across all presentation types: *n* = 12 (5.7%) for Combined, *n* = 20 (9.6%) for Inattentive, and *n* = 0 for the Hy/Imp presentation (Fig. 1; Table 2).

**Table 2:** Demographic table of the study population by study ADHD diagnoses

**CATEGORICAL VARIABLES:**		Total *N* = 645	Total ADHD *N* = 213	Non-ADHD *N* = 432	Combined *N* = 98	Inattentive *N* = 100	Hy/Imp *N* = 15	
Demographic factors	Description	Col % (*N*)	Col % (*N*)	Col % (*N*)	Col % (*N*)	Col % (*N*)	Col % (*N*)	*P*-value^1^
**CHARGE** **(Early Childhood)** **Final Diagnosis**	**TD**	32.4 (209)	15.0 (32)	41.0 (177)	12.2 (12)	20.0 (20)	—^11^	< 0.0001
	**OEC**	15.3 (99)	16.9 (36)	14.6 (63)	14.3 (14)	21.0 (21)	—	
	**DD**	13.0 (84)	13.2 (28)	13.0 (56)	16.3 (16)	10.0 (10)	—	
	**Autism**	39.2 (253)	54.9 (117)	31.5 (136)	57.1 (56)	49.0 (49)	80.0 (12)	
**Race** ^**3**^	**White**	51.6 (333)	48.8 (229)	53.0 (229)	54.1 (53)	45.0 (45)	40.0 (6)	0.74
	**Black**	3.7 (24)	3.8 (16)	3.7 (16)	—	—	—	
	**Asian**	4.8 (31)	3.8 (23)	5.3 (23)	—	—	—	
	**Hispanic**	27.4 (177)	31.5 (110)	25.5 (110)	24.5 (24)	37.0 (37)	40.0 (6)	
	**Multi-racial**	11.9 (77)	11.3 (53)	12.3 (53)	12.2 (12)	10.0 (10)	—	
**Sex**	**Female**	24.2 (156)	20.7 (44)	25.9 (112)	17.4 (17)	24.0 (24)	—	0.34
	**Male**	75.8 (489)	79.3 (169)	74.1 (320)	82.7 (81)	76.0 (76)	80.0 (12)	
**Parents’ Max Education**	**High school or less**	5.7 (37)	7.0 (15)	5.1 (22)	—	9.0 (9)	—	0.29
	**College**	30.5 (197)	36.6 (78)	27.6 (119)	36.7 (36)	38 (38)	—	
	**Bachelor**	39.4 (254)	33.8 (72)	42.1 (182)	35.7 (35)	30.0 (30)	46.7 (7)	
	**Graduate**	24.3 (157)	22.5 (48)	25.2 (109)	2.5 (22)	23.0 (23)	—	
**Parents ADHD** ^**2**^	**Yes**	9.3 (60)	14.6 (31)	6.7 (29)	20.4 (20)	9.0 (9)	—	0.0004
	**No**	90.7 (585)	85.4 (182)	93.3 (403)	79.6 (78)	91.0 (91)	86.7 (13)	
**Mother Pregnancy Smoking** ^**4**^	**Yes**	9.9 (64)	11.7 (25)	9.0 (39)	9.2 (9)	14.0 (14)	—	0.27
	**No**	83.4 (538)	79.3 (169)	85.4 (369)	81.6 (80)	76.0 (76)	86.7 (13)	
	**Missing**	6.7 (43)	8.9 (19)	5.6 (24)	9.2 (9)	10.0 (10)	—	
**Age at ADHD assessment**	**8 to 10**	27.0 (174)	32.9 (70)	24.1 (104)	39.8 (39)	25.0 (25)	40.0 (6)	0.045
	**11 to 13**	18.0 (116)	17.4 (37)	18.3 (79)	18.4 (18)	18.0 (18)	46.7 (7)	
	**14 to 17**	38.1 (246)	38.0 (81)	38.2 (165)	32.7 (32)	42.0 (42)	46.7 (7)	
	**18 to 20**	16.9 (109)	11.7 (25)	19.4 (84)	9.2 (9)	15.0 (15)	—	
**Mother Metabolic Condition during pregnancy**	**No Diabetes**	78.9 (509)	73.7 (157)	81.5 (352)	76.5 (75)	73.0 (73)	60.0 (9)	0.04
	**Diabetes**	16.7 (108)	21.1 (45)	14.6 (63)	19.4 (19)	20.0 (20)	40.0 (6)	
	**Missing**	4.3 (28)	5.2 (11)	3.9 (17)	—	7.0 (7)	—	
**Payment for Delivery** ^**5**^	**Government or No Insurance**	15.0 (97)	16.4 (35)	14.4 (62)	13.3 (13)	20.0 (20)	—	0.35
	**Private Insurance**	83.4 (538)	80.8 (172)	84.7 (366)	83.7 (82)	77.0 (77)	86.7 (13)	
**Regional Center (RC)**	**Northern Calif RC’s**	50.1 (323)	49.8 (106)	50.2 (217)	53.1 (52)	45.0 (45)	60.0 (9)	0.59
	**Bay and coastal RC’s**	31.8 (205)	31.9 (68)	31.7 (137)	31.6 (31)	33.0 (33)	—	
	**Central Valley RC’s**	18.1 (117)	18.3 (39)	18.1 (78)	15.3 (15)	22.0 (22)	—	
**Preterm Birth**	**Yes**	14.9 (96)	13.6 (29)	15.5 (67)	12.2 (12)	16.0 (16)	—	0.67
	**No**	85.1 (549)	86.4 (184)	84.5 (365)	87.8 (86)	84.0 (84)	93.3 (14)	
**CONTINUOUS VARIABLES**		**Total** *N* = 645	**Any ADHD *N*** = **213**	**Non-ADHD** *N* = 432	**Combined** *N* = 98	**Inattentive** *N* = 100	**Hy/Imp** *N* = 15	
		**Mean (STD)**	**Mean (STD)**	**Mean (STD)**	**Mean (STD)**	**Mean (STD)**	**Mean (STD)**	**P-value**
**Age at Early Childhood Clinical Assessment**	**Years**	3.76 (0.03)	3.82 (0.06)	3.72 (0.04)	3.80 (0.09)	3.85 (0.08)	3.82 (0.22)	0.53
**Mother age at Delivery**	**Years**	31.60 (0.2)	31.25 (0.35)	31.76 (0.28)	30.93 (0.50)	31.58 (0.55)	31.17 (1.07)	0.60
**Child gestational age**	**Weeks**	38.79 (0.1)	38.98 (0.15)	38.69 (0.15)	39.16 (0.21)	38.74 (0.22)	39.34 (0.48)	0.41
**Birthweight T score** ^**6**^		49.10 (0.4)	49.00 (0.74)	49.14 (0.55)	50.02 (1.08)	48.00 (1.11)	48.97 (2.53)	0.65
**Early Childhood Mullen**	**Early Learning Comp DQ**	8.39 (2.66)	7.90 (2.28)	8.62 (2.8)	8.17 (2.24)	7.97 (2.19)	5.68 (2.06)	< 0.0001
**8 to 20 years old Study Stanford Binet**	**AB IQ**	95.0 (1.0)	91.28 (1.82)	96.58 (1.25)	90.72 (2.84)	93.20 (2.19)	81.63 (11.24)	0.054
**ADHD Symptoms Measurements**								
**Early Childhood ABC**	**Hyperactivity – Subscale 4** ^**7**^	11.17 (0.5)	16.43 (0.86)	8.72 (0.50)	20.29 (1.31)	12.28 (1.09)	18.69 (2.61)	< 0.0001
	**Hyperactivity and impulsivity items** ^**8**^	6.92 (0.3)	10.30 (0.56)	5.32 (0.34)	12.79 (0.86)	7.63 (0.72)	12.38 (1.59)	< 0.0001
	**Inattention items** ^**9**^	2.26 (0.1)	3.22 (0.17)	1.80 (0.10)	3.88 (0.26)	2.42 (0.22)	4.20 (0.65)	< 0.0001
	**Oppositionality and defiance items** ^**10**^	2.04 (0.1)	2.91 (0.17)	1.63 (0.09)	3.53 (0.26)	2.35 (0.23)	2.73 (0.59)	< 0.0001
**8 to 20 years old Study Conners**	**Conners-3-P Inattention T-Score**	58.76 (0.8)	76.60 (1.00)	52.01 (0.69)	79.26 (1.28)	75.65 (1.41)	65.00 (4.56)	< 0.0001
	**Conners-3-P Hy/Imp T-Score**	58.11 (0.9)	75.85 (1.41)	51.40 (0.68)	82.85 (1.49)	67.26 (2.03)	82.71 (3.03)	< 0.0001

^1^P-values are based on Chi-squared tests for comparison of categorical variables, and one-way ANOVA for comparison of continuous measures. These are tests of differences across the mutually exclusive ADHD categories (Non-ADHD, Combined, Inattentive, and Hyperactive/Impulsive)

^2^At least one parent endorsed having a history of ADHD by self-report survey, the “Yes” category is combined from “Yes-Not Specified”, “Yes-Possible”, and “Yes-Diagnosed”

^3^There are 3 participants who had missing information on race/ethnicity

^4^Yes values are mothers who smoked any tobacco product either before or during pregnancy. Note that there are only a handful of non-cigarette products reported. This does NOT include non-smoked tobacco products such as snuff and chew, or nicotine patches. No e-cigarettes reported so far because children in the early, Time Point 1 study were mostly born before e-cigarettes became popular

^5^There are 10 participants who had missing information on Child Delivery Payment Method

^6^Adjusted by gestational age [48]

^7^ Score range 0–48. Sum of ABC item #s 1, 7, 13, 15, 18, 21, 24, 28, 31, 38, 39, 44, 48, 51, 54, 56

^8^ Score range 0–30. Sum of ABC item #s 1, 7, 13, 15, 21, 31, 38, 39, 48, 54

^9^ Score range 0–9. Sum of ABC item #s: 28, 44, 51

^10^ Score range 0–9. Sum of ABC item #s: 18, 24, 56

^11^ Cells with size ≤ 5 were suppressed

Table 2 also shows the bivariate analyses of ADHD diagnoses. We found a strong association between an autism diagnosis in early childhood and an ADHD diagnosis in mid-childhood through adolescence (*p* < 0.0001). Having parents with a self-reported ADHD diagnosis history was also strongly predictive of a higher risk for the child to develop ADHD (*p* = 0.0004), whereas older age at the time of assessment predicted a lower risk of ADHD (*p* = 0.045). Most sociodemographic, neonatal, health, and other characteristics (e.g., race, delivery payment method, child gestational age, child birthweight T-score, maternal smoking) did not demonstrate significance in the bivariate analyses with ADHD.

For the multivariate-adjusted Poisson regression models, we assessed model assumptions: absence of overdispersion and no collinearity. Both assumptions were confirmed. The first block of Table 3 shows the findings from multivariate models for the association between the CHARGE early childhood developmental group and ADHD Combined-Hy/Imp presentation type at ages 8–21. Children with autism, DD, or OEC were much more likely to have ADHD Combined-Hy/Imp presentations compared to the TD group: RR = 5.35, 95% CI [3.04, 9.42] for autism; RR = 4.43 [2.32, 8.44] for DD; RR = 3.06 [1.54, 6.06] for OEC. Females were less likely to have ADHD Combined-Hy/Imp presentations than males (RR = 0.68 [0.44, 1.03]). The older age groups were less likely to have ADHD Combined-Hy/Imp presentations compared with 8- to 10-year-olds: RR = 0.72 [0.47, 1.11] for 11- to 13-year-olds; RR = 0.73 [0.51, 1.05] for 14- to 16-year-olds; RR = 0.36 [0.19, 0.67] for 17- to 20-year-olds. Refitting age into the model as a continuous variable showed about a 7% decrease in risk for every year older (RR = 0.93 [0.89, 0.97]; Appendix 6). Children whose parents reported having an ADHD history were far more likely to have ADHD Combined-Hy/Imp presentations than those whose parents reported no ADHD history (RR = 2.07 [1.41, 3.04]). Maternal metabolic conditions, specifically diabetes (pre-existing and gestational), raised the risk of Combined-Hy/Imp presentations, though the results were rather imprecise (RR = 1.27 [0.89, 1.80]). No significant findings regarding race/ethnicity, regional center that managed services, parents’ education, or health insurance status at delivery from the model emerged.

**Table 3:** Result of multivariate Poisson log model for outcome variables as combined, hy/imp, and any ADHD by 20 imputation (Blank blocks indicates the variables were not selected)

Parameter Description	ADHD Combined-Hy/Imp Model			ADHD Inattentive Model			Any ADHD Presentations		
	RRs	95% CI Mean	*P*-value	RRs	95% CI Mean	*P*-value	RRs	95% CI Mean	*P*-value
**Early Diagnostic Group**									
TD	Reference	.	.	Reference	.	.	Reference	.	.
OEC	3.06	1.54, 6.06	0.001	2.56	1.40, 4.16	0.002	2.39	1.59, 3.59	< 0.001
DD	4.43	2.32, 8.44	< 0.001	1.44	0.71, 2.94	0.32	2.37	1.55, 3.64	< 0.001
Autism	5.35	3.04, 9.42	< 0.001	2.56	1.57, 4.17	< 0.001	3.12	2.22, 4.38	< 0.001
**Sex**									
Male	Reference	.	.	Reference	.	.	Reference	.	.
Female	0.68	0.44, 1.03	0.07	1.13	0.55, 2.31	0.75	0.81	0.62, 1.05	0.11
**MINI/DISC Age**									
8–10 yrs old	Reference	.	.	Reference	.	.	Reference	.	.
11–13 yrs old	0.72	0.47, 1.11	0.14	0.89	0.46, 1.74	0.74	0.86	0.63, 1.17	0.34
14–16 yrs old	0.73	0.51, 1.05	0.09	1.17	0.69,1.98	0.57	0.88	0.69, 1.13	0.31
17–20 yrs old	0.36	0.19, 0.67	0.001	0.82	0.43, 1.55	0.53	0.55	0.38, 0.80	0.002
**Sex – Age Interaction**									
Female 11–13 yrs				1.37	0.43,4.40	0.59			
Female 14–16 yrs				0.69	0.26, 1.79	0.44			
Female 17–20 yrs				0.29	0.04, 2.32	0.24			
**Parents ADHD**									
No	Reference	.	.				Reference	.	.
Yes	2.02	1.38, 2.97	< 0.001				1.58	1.18, 2.10	0.002
**Mother Metabolic**									
No Diabetes	Reference	.	.				Reference	.	.
Diabetes	1.26	0.88, 1.81	0.20				1.18	0.92, 1.51	0.18
**Parents Maximum Education**									
Graduate				Reference	.	.			
Bachelor				0.80	0.49, 1.30	0.36			
Some college				1.30	0.82, 2.05	0.26			
High School or Less				1.64	0.83, 3.23	0.16			

The second block of Table 3 shows multivariate-adjusted results on the association between CHARGE diagnostic group and ADHD Inattentive presentations at ages 8–20. When comparing the Inattentive to the Combined and Hy/Imp presentation model, only the early childhood study diagnostic group retained statistical significance as an effect. In this model, autistic children and OEC were significantly more likely to receive an ADHD Inattentive diagnosis than those in the TD group (RR = 2.56 [1.57, 4.17] for autism; RR = 2.56 [1.40, 4.16] for OEC). Only the DD group did not significantly differ from TD in their risk of having an ADHD Inattentive presentation (RR = 1.44 [0.71, 2.94], *p* = 0.32). Besides adjusting for sex and age, the only additional covariate retained in the model based on the LRT < 0.2 was parents’ maximum education. A nonsignificant trend suggests lower parental maximum education may be associated with greater risk for a diagnosis of Inattentive ADHD. An interaction in the continuous age model showed that Inattentive ADHD risk remained stable with age in males (RR = 1.0 \[0.95, 1.06]) but declined in females (RR = 0.92 \[0.84, 1.02]), suggesting sex-specific effects of aging (Appendix 6). This interaction was not seen in the other two ADHD models.

The third block of Table 3 presents the model for any ADHD presentation as the outcome. The variables retained in this model are the same as those in the ADHD Combined or Hy/Imp model, but the magnitude of all associations is attenuated, while the estimates demonstrate substantially greater precision.

**Fig. 2:**
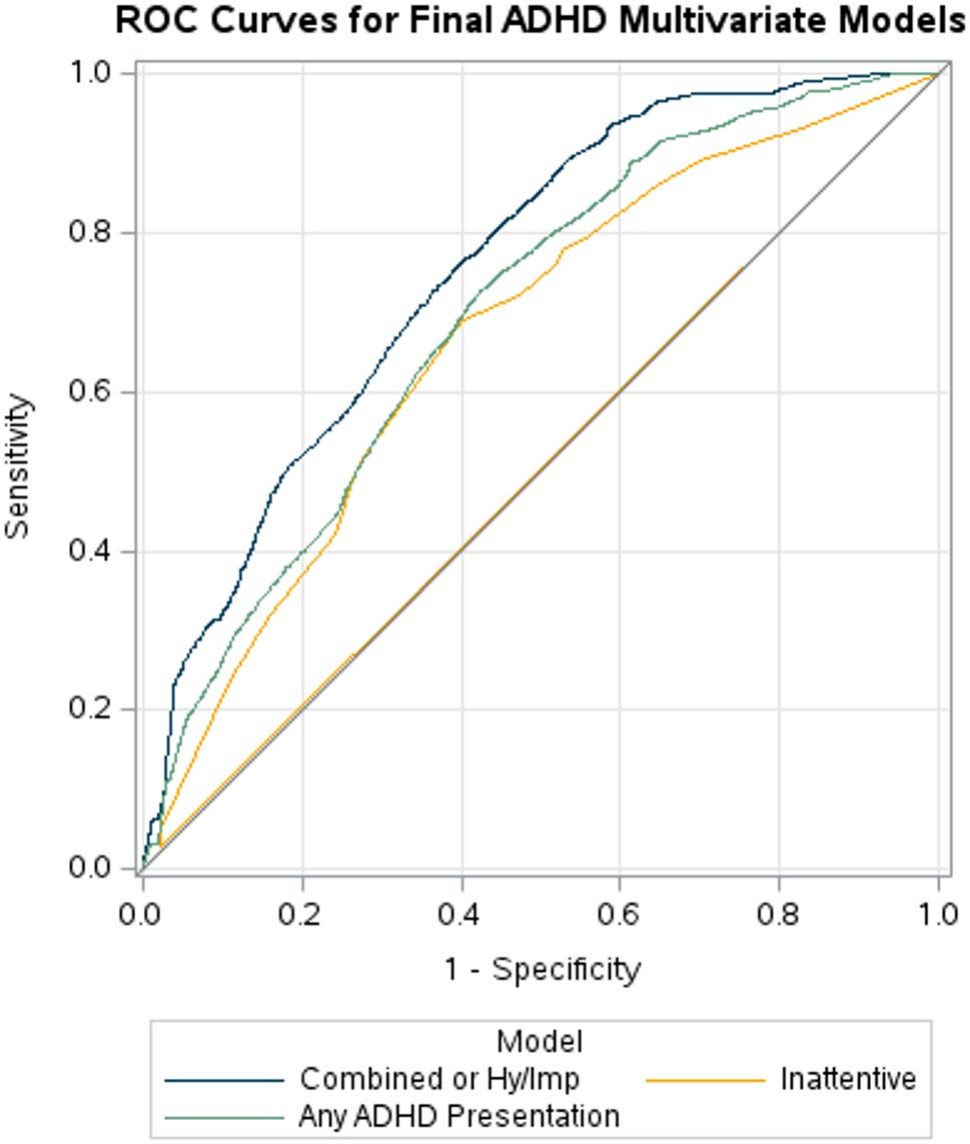
ROC curve for the selected multivariate logistic regression on outcomes ADHD Combined-Hy/Imp presentation (AUC = 0.77), Inattentive presentation (AUC = 0.69), and Any ADHD presentation (AUC = 0.71)

An ROC curve compares the true positive rate (sensitivity) with the false positive rate (1 − specificity) across varying thresholds, plotted within the unit square. The ROC curves for the Combined-Hy/Imp ADHD model, Inattentive ADHD model, and Any ADHD presentation model yielded areas under the curve (AUC) of 0.77, 0.69, and 0.71, respectively, indicating all three hover around the suggested cut-off point of 0.7 between acceptable and low discrimination (Fig. 2) [24].

According to clinical interviews (MINI/DISC) depression/dysthymia was present in 13.0% of participants, highest in the OEC group (29.3%) versus autism (9.9%), TD (11.5%), and DD (7.1%) (Appendix 2). Generalized anxiety affected 15.2%, again highest in OEC (22.2%) versus autism (15.4%), DD (16.7%), and TD (11.0%). Rates were lower for obsessive-compulsive disorder (6.5%), oppositional defiant disorder (8.5%), posttraumatic stress disorder (1.1%), and conduct disorder (1.4%). Learning disabilities were reported in 26.7% of the total cohort (Appendix 2), with the highest rates in DD (44.0%) and autism (38.7%), and lower rates in OEC (24.2%) and TD (6.2%). At the assessment, 11.6% were taking ADHD medication—5.6% on stimulants and 2.3% on nonstimulants.

## Discussion

This study demonstrates a strong association between early childhood neurodevelopmental diagnostic group (ages 2–5 years) and later ADHD diagnoses and presentation types at ages 8–20 years, particularly in autistic children. Stronger associations were observed for the Combined and Hy/Imp presentations than for the Inattentive presentation. These findings underscore the need to screen for ADHD symptoms in autistic children and other neurodevelopmental conditions, using tools suited to this population, especially in the context of intellectual ability, such as the ABC-2nd edition [25], and the teacher SAID [21], and parent version, which is under development. Ultimately, broader normative assessment tools are needed specifically for these populations [26, 27]. The high prevalence of ADHD in autistic children, DD, and other early concerns also highlights the need for ADHD strategies targeting impulsivity, inattention, and hyperactivity symptoms in populations with neurodevelopmental conditions. Our findings suggest prioritizing interventions for Combined and Inattentive presentations, as the Hy/Imp type was rare and less stable over time, consistent with prior research [28]. The small number of children/adolescents with the Hy/Imp phenotype precluded analysis of those diagnoses alone, and therefore we presented results for the group that included Combined/Hy/Imp, where Combined indicates participants who met criteria for both the Inattentive and the Hy/Imp subtypes. Our data, however, are consistent with longitudinal studies [28] finding the Hy/Imp presentation tends to wane with maturity, with the Hy/Imp declining during middle and late childhood. Our study population provided an opportunity to conduct a preliminary exploration of Hy/Imp in the autism and DD cohorts that confirmed the earlier findings.

Our finding that early childhood autism diagnosis strongly predicts later ADHD diagnosis extends previous cross-sectional findings on symptoms [3]. ADHD rates were highest in autistic children during early childhood and remained elevated in mid-childhood and adolescence when diagnoses are more reliable. These findings align with studies showing persistence of ADHD from childhood to adolescence in non-autistic populations [3, 29, 30] and high autism–ADHD comorbidity [31–33]. Though explanatory models are reviewed elsewhere [34], our results may support developmental risk or mutual influence [35–39].

Autism diagnosis significantly predicted all ADHD presentations, while male sex, parental ADHD history, and younger age at assessment predicted Combined/Hy/Imp but not Inattentive type. ROC analysis showed moderate discrimination for the Combined/Hy/Imp model (AUC = 0.77) and low for the Inattentive model (AUC = 0.69), suggesting other factors (e.g., ODD) may contribute to the latter [40]. Notably, the OEC group was strongly associated with the Inattentive presentation. Although these children did not meet diagnostic criteria at our clinical evaluation, many were initially labeled with autism or DD, raising the possibility that early inattention may be misinterpreted as autism or DD. This finding suggests that early symptoms or precursors of the Inattentive ADHD presentation type may be misinterpreted as symptoms of autism or DD, highlighting the importance of a comprehensive evaluation given the ubiquitous nature of non-pathognomonic symptoms (i.e., inattention, social deficits, etc.) common to many disorders.

Subtype instability and decline in Hy/Imp symptoms with age are reflected in our data [28, 31, 41, 42], with a drop in Combined-Hy/Imp presentations from ages 8–10 to 18–20 years. This highlights the need for longitudinal research on ADHD subtype trajectories. Finally, the TD group had a higher-than-expected ADHD rate (15.3% vs. general population estimate of 12.9% [43]), possibly due to overrepresentation of males (matching autism sex ratios) and selective retention of families with concerns.

We assessed for the presence of other psychiatric disorders in addition to ADHD and found high rates of depression, anxiety, oppositional defiant disorder, and other common childhood conditions, consistent with the known high ADHD comorbidity [28, 44, 45]. Notably, the OEC group showed surprisingly high rates of depression and anxiety, warranting further study. Parents also reported high rates of current or past learning disabilities, though these were not independently verified due to study scope and participant burden. Misinterpretation of the term “learning disability” may have contributed to overreporting.

**Strengths and Limitations.** Although parents reported on school-related symptoms and impairment, teacher ratings were not obtained due to resource constraints. A large subset of participants with intellectual and adaptive impairments were excluded, as the clinical interviews were not designed to validly assess ADHD symptoms in this group, potentially introducing bias if excluded individuals differed in symptom profiles. Additionally, 11.36% of participants were prescribed ADHD medications, reflecting the prevalence of symptoms; however, medication use may have reduced symptom reporting. Most were prescribed stimulants with short half-lives, so parents likely still observed symptoms. The OEC and Hy/Imp groups were small and heterogeneous, limiting statistical power and subgroup estimate reliability. Finally, although the broad age range for follow-up had advantages, each participant was assessed only once, limiting insight into developmental changes relevant to ADHD.

Several strengths of the study include the large epidemiological sample size; the population-based enrollment from community-diagnosed autism and DD cases; and the population-based sampling of controls from births in the Vital Statistics records, matched by age, sex, and region. This approach resulted in a highly diverse overall study population of approximately 52% White non-Hispanic, 27% Hispanic, and 21% Black, Asian, and multi-racial participants. Additionally, the diagnoses—for both early childhood neurodevelopmental groups and for ADHD at older ages—were based on extensive diagnostic assessments and multiple measures administered by trained clinicians interacting with the children/adolescents and their parents using standardized protocols. Moreover, because we covered a wide age range, we were able to detect differences between mid/late childhood and adolescence. Given that overlapping symptom presentations—such as parent-reported inattention—can obscure clinical distinctions, particularly in younger children or those with lower cognitive functioning [46], future studies should incorporate multi-method assessment strategies.

This study highlights the importance of evaluating ADHD in autistic children and other DD, who show significantly higher rates of ADHD than their TD peers. Inattentive symptoms—alone or as part of the Combined presentation—were most common, aligning with prior findings that inattention is prevalent across neurodevelopmental conditions [3, 46]. Thus, targeted assessment tools and interventions for inattention are especially important for these groups. While existing approaches (e.g., behavioral management, social skills training, medication) exhibit promise for co-occurring ADHD and autism, empirical evidence remains limited [47]. Novel, tailored interventions are needed for those with significant ADHD symptoms in the context of autism and/or DD. It remains unclear whether ADHD symptoms in youth with both autism and DD differ from those with DD alone, underscoring the need for future research on their presentation and developmental trajectories associated with comorbid ADHD.

## Electronic supplementary material

Below is the link to the electronic supplementary material.

Supplementary Material 1

## Data Availability

Data are available from the authors upon request.
