# Supplementary material for: Predicting later ADHD presentation types from early childhood autism and intellectual disability
Source: Eur Child Adolesc Psychiatry. 2025 Jul 17;34(12):4023–33. doi: 10.1007/s00787-025-02805-7 (PMC12743070; doi:10.1007/s00787-025-02805-7)
Supplement: Supplementary file 1 — Supplementary Material 1 [file 787_2025_2805_MOESM1_ESM.docx]

**APPENDICES**

Appendix 1. Table of Variable Missingness*

| Description | N | Missing Number | Missing Percentage |
| --- | --- | --- | --- |
| Early Diagnostic Group | 645 | 0 | 0 |
| Age of mother at time of child’s birth | 645 | 0 | 0 |
| Parents' maximum education level | 645 | 0 | 0 |
| Regional Center catchment area group at birth | 645 | 0 | 0 |
| Vineland Composite Developmental Quotient | 645 | 0 | 0 |
| Child Age at MINI/DISC Completion (8-9, 10-14, 15-21) | 645 | 0 | 0 |
| MINI/DISC ADHD Diagnosis | 645 | 0 | 0 |
| Early Learning Composite: Developmental Quotient | 644 | 1 | 0.2 |
| Child's Race/Ethnicity | 642 | 3 | 0.5 |
| Gestational age in Week | 640 | 5 | 0.8 |
| Birthweight adjusted fir gestational age | 639 | 6 | 0.9 |
| Payment method for delivery of child | 635 | 10 | 1.6 |
| Early ABC | 618 | 27 | 4.2 |
| Maternal Metabolic Condition | 617 | 28 | 4.3 |
| Mother Smoked any Tobacco Product before or during pregnancy | 602 | 43 | 6.7 |
| Mother with ADHD history | 591 | 54 | 8.4 |
| Father with ADHD history | 591 | 54 | 8.4 |
| Later ABC | 458 | 187 | 29 |
| Stanford Binet | 389 | 256 | 39.7 |
| Conners’ | 355 | 290 | 45 |

*All variables were included in the imputation models except Later ABC, Stanford Binet and Conners’. Notably, the ABC and Conners’ were excluded because of redundancy/overlap with the outcomes of interest.

Appendix 2. The Prevalence of Psychiatric Diagnoses, Learning Disabilities, and ADHD Medication Use in the ReCHARGE Study Across CHARGE Study Diagnostic Groups

|  | Autism (N=253) | DD  (N=84) | OEC  (N=99) | TD  (N=209) | Total  (N=645) |
| --- | --- | --- | --- | --- | --- |
| Major Depression/ Dysthymia | 25 (9.9%) | 6 (7.1%) | 29 (29.3%) | 24 (11.5%) | 84 (13.0%) |
| Obsessive Compulsive Disorder | 26 (10.3%) | 4 (4.8%) | 8 (8.1%) | 4 (1.9%) | 42 (6.5%) |
| Posttraumatic Stress Disorder | 3 (1.2%) | 0 (0.0%) | 1 (1.0%) | 3 (1.4%) | 7 (1.1%) |
| Conduct Disorder | 4 (1.6%) | 3 (3.6%) | 1 (1.0%) | 1 (0.5%) | 9 (1.4%) |
| Oppositional Defiant Disorder | 24 (9.5%) | 8 (9.5%) | 12 (12.1%) | 11 (5.3%) | 55 (8.5%) |
| Generalized Anxiety Disorder | 39 (15.4%) | 14 (16.7%) | 22 (22.2%) | 23 (11.0%) | 98 (15.2%) |
| Learning Disability | 98 (38.7%) | 37 (44.0%) | 24 (24.2%) | 13 (6.2%) | 172 (26.7%) |
| Any Current ADHD Medication | 41 (16.2%) | 12 (14.3%) | 9 (9.1%) | 13 (6.2%) | 75 (11.6%) |
| Current Nonstimulant ADHD Medication | 8 (3.2%) | 3 (3.6%) | 2 (2.0%) | 2 (1.0%) | 15 (2.3%) |
| Current Stimulant ADHD Medication | 21 (8.3%) | 7 (8.3%) | 5 (5.1%) | 3 (1.4%) | 36 (5.6%) |

Appendix 3. Directed Acyclic Graph (DAG)


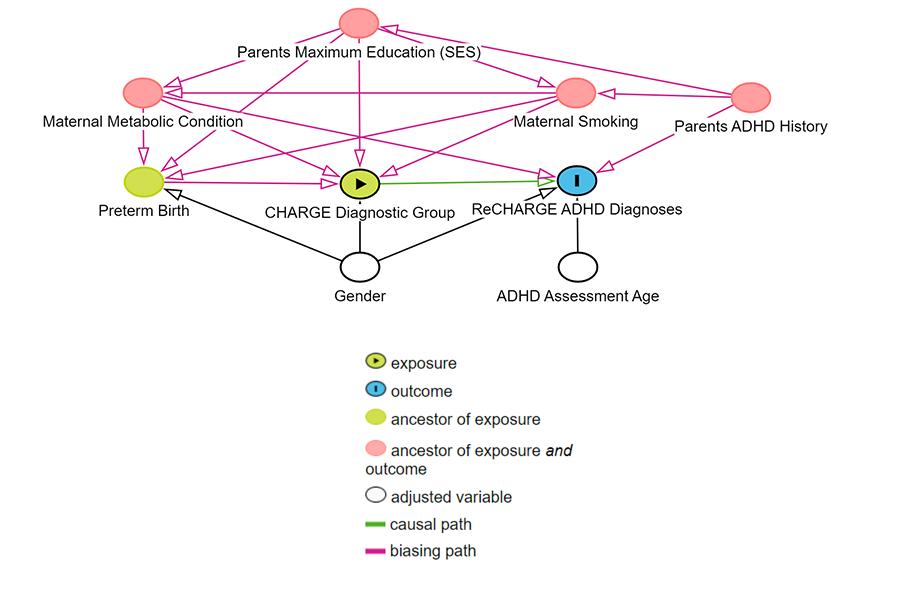


Appendix 4. Univariate Poisson Log Regression Model Result for Three ADHD Outcomes (Combined/Hy/Imp, Inattentive, and All ADHD)

|  |  | ADHD Combined/Hy/Imp | | ADHD Inattentive | | Total ADHD | |
| --- | --- | --- | --- | --- | --- | --- | --- |
| Description |  | Relative  Risk | P-value | Relative Risk | P-value | Relative Risk | P-value |
| Early Diagnostic Group | TD | 1 | . | 1 | . | 1 | . |
|  | OEC | 3.70 | <0.001 | 2.38 | 0.004 | 2.63 | <0.001 |
|  | DD | 6.74 | <0.001 | 1.51 | 0.341 | 2.89 | <0.001 |
|  | ASD | 7.31 | <0.001 | 2.68 | 0.003 | 3.62 | <0.001 |
| Sex | Female | 0.62 | 0.023 | 0.92 | 0.663 | 0.79 | 0.07 |
| Age at MINI Interview | 8-10 yrs old | 1 | . | 1 | . | 1 | . |
|  | 11-13 yrs old | 0.64 | 0.06 | 0.96 | 0.88 | 0.79 | 0.16 |
|  | 14-16 yrs old | 0.63 | 0.02 | 1.05 | 0.84 | 0.82 | 0.12 |
|  | 17-20 yrs old | 0.35 | 0.001 | 0.78 | 0.41 | 0.57 | 0.005 |
| Mullen Composite DQ | 10 Per Unit | 1.07 | 0.10 | 1.01 | 0.771 | 1.04 | 0.154 |
| Birthweight T-score | Adjusted by gestational age | 1.01 | 0.06 | 1.00 | 0.69 | 1.00 | 0.46 |
| Preterm Birth | Yes | 0.66 | 0.13 | 0.81 | 0.382 | 0.75 | 0.07 |
| Parent ADHD | Either Mother or Father ADHD | 2.23 | <0.001 | 1.26 | 0.461 | 1.66 | 0.001 |
| Metabolic Conditions | No Diabetes | 1 | . | 1 | . | 1 | . |
|  | Diabetes | 1.36 | 0.09 | 0.81 | 0.32 | 1.21 | 0.12 |
| Mother Smoking | Smoking before or during pregnancy | 0.90 | 0.72 | 1.14 | 0.62 | 1.01 | 0.96 |
| Mother Age | By years | 0.99 | 0.49 | 1.01 | 0.35 | 1 | 0.998 |
| Child Race | White + Asian | 1 | . | 1 | . | 1 | . |
|  | Black | 0.85 | 0.63 | 0.77 | 0.63 | 0.87 | 0.60 |
|  | Hispanic | 1.05 | 0.78 | 1.34 | 0.14 | 1.15 | 0.26 |
|  | Multi-racial | 1.00 | 0.997 | 0.87 | 0.67 | 0.96 | 0.82 |
| Parent's max education | Graduate | 1 | . | 1 | . | 1 | . |
|  | Bachelor | 1.00 | 0.99 | 0.81 | 0.40 | 0.95 | 0.71 |
|  | College | 1.28 | 0.30 | 1.24 | 0.43 | 1.21 | 0.23 |
|  | High school or less | 0.92 | 0.83 | 1.50 | 0.27 | 1.18 | 0.48 |
| Payment Delivery | Using private insurance at birth | 1.36 | 0.22 | 0.83 | 0.44 | 1.05 | 0.75 |
| Regional Center | Regional Center 1 | 1 | . | 1 | . | 1 | . |
|  | Regional Center 2 | 1.06 | 0.76 | 1.21 | 0.39 | 1.11 | 0.42 |
|  | Regional Center 3 | 0.93 | 0.77 | 1.10 | 0.65 | 0.99 | 0.94 |

Appendix 5. Full Multivariate Poisson Log Regression Model Result for Three ADHD Outcomes (Combined/Hy/Imp, Inattentive, and All ADHD)

|  |  | ADHD Combined/Hy/Imp | | ADHD Inattentive | | Total ADHD | |
| --- | --- | --- | --- | --- | --- | --- | --- |
| Description |  | Relative  Risk | P-value | Relative Risk | P-value | Relative Risk | P-value |
| Early Diagnostic Group | TD | 1 | . | 1 | . | 1 | . |
|  | OEC | 3.73 | <0.001 | 2.36 | 0.005 | 2.61 | <0.001 |
|  | DD | 6.77 | <0.001 | 1.52 | 0.33 | 2.88 | <0.001 |
|  | ASD | 6.94 | <0.001 | 2.65 | 0.002 | 3.55 | <0.001 |
| Sex | Female | 0.62 | 0.027 | 0.93 | 0.72 | 0.79 | 0.09 |
| Age at MINI Interview | 8-10 yrs old | 1 | . | 1 | . | 1 | . |
|  | 11-13 yrs old | 0.69 | 0.097 | 0.94 | 0.84 | 0.84 | 0.28 |
|  | 14-16 yrs old | 0.70 | 0.075 | 1.06 | 0.80 | 0.88 | 0.34 |
|  | 17-20 yrs old | 0.36 | 0.002 | 0.69 | 0.21 | 0.55 | 0.002 |
| Mullen Composite DQ | 10 Per Unit | 1.06 | 0.124 | 1.02 | 0.73 | 1.04 | 0.17 |
| Birthweight T-score | Adjusted by gestational age | 1.01 | 0.054 | 1.00 | 0.71 | 1.00 | 0.40 |
| Preterm Birth | Yes | 0.65 | 0.105 | 0.83 | 0.46 | 0.76 | 0.10 |
| Parent ADHD | Either Mother or Father ADHD | 2.15 | <0.001 | 1.25 | 0.48 | 1.62 | 0.002 |
| Metabolic Conditions | No Diabetes | 1 | . | 1 | . | 1 | . |
|  | Diabetes | 1.42 | 0.059 | 1.27 | 0.27 | 1.24 | 0.08 |
| Mother Smoking | Smoking before or during pregnancy | 0.91 | 0.735 | 1.16 | 0.60 | 1.01 | 0.96 |
| Mother Age | By years | 0.99 | 0.368 | 1.01 | 0.42 | 1.00 | 0.87 |
| Child Race | White + Asian | 1 | . | 1 | . | 1 | . |
|  | Black | 0.82 | 0.558 | 0.82 | 0.72 | 0.88 | 0.65 |
|  | Hispanic | 1.02 | 0.915 | 1.35 | 0.13 | 1.15 | 0.27 |
|  | Multi-racial | 1.02 | 0.946 | 0.88 | 0.71 | 0.98 | 0.90 |
| Parent's max education | Graduate | 1 | . | 1 | . | 1 | . |
|  | Bachelor | 0.97 | 0.896 | 0.79 | 0.36 | 0.93 | 0.60 |
|  | College | 1.23 | 0.376 | 1.20 | 0.51 | 1.18 | 0.30 |
|  | High school or less | 0.93 | 0.869 | 1.45 | 0.31 | 1.17 | 0.51 |
| Payment Delivery | Using private insurance at birth | 1.41 | 0.179 | 0.83 | 0.43 | 1.05 | 0.73 |
| Regional Center | Regional Center 1 | 1 | . | 1 | . | 1 | . |
|  | Regional Center 2 | 1.10 | 0.617 | 1.21 | 0.40 | 1.12 | 0.38 |
|  | Regional Center 3 | 0.96 | 0.852 | 1.11 | 0.62 | 1.00 | 0.99 |

Appendix 6. Result of Multivariate Poisson Log Model for Outcome Variables as Combined, Hy/Imp, and Any ADHD by 20 Imputation (Treating Age as Continuous variable)

| **Parameter Description** | **ADHD Combined or Hyperactive/Impulsive Model** | | | **ADHD Inattentive Model** | | | **Any ADHD Presentations** | | | |
| --- | --- | --- | --- | --- | --- | --- | --- | --- | --- | --- |
|  | RRs | 95% CI Mean | P-value | RRs | 95% CI Mean | P-value | RRs | 95% CI Mean | P-value |  |
| **Early Diagnostic Group** |  |  |  |  |  |  |  |  |  |  |
| TD | Reference | . | . | Reference | . | . | Reference | . | . |  |
| OEC | 3.00 | 1.51, 5.96 | 0.002 | 2.41 | 1.39, 4.19 | 0.002 | 2.37 | 1.58, 3.57 | <0.001 |  |
| DD | 4.34 | 2.28, 8.29 | <0.001 | 1.44 | 0.70, 2.93 | 0.32 | 2.33 | 1.52, 3.57 | <0.001 |  |
| ASD | 5.24 | 2.98, 9.21 | <0.001 | 2.45 | 1.50, 4.01 | <0.001 | 3.05 | 2.17, 4.29 | <0.001 |  |
| **Sex** |  |  |  |  |  |  |  |  |  |  |
| Male | Reference | . | . | Reference | . | . | Reference | . | . |  |
| Female | 0.67 | 0.44, 1.03 | 0.06 | 0.88 | 0.58, 1.35 | 0.56 | 0.81 | 0.62, 1.05 | 0.12 |  |
| **MINI/DISC Age** |  |  |  |  |  |  |  |  |  |  |
| Year | 0.93 | 0.89, 0.97 | 0.001 | 1.00 | 0.95, 1.06 | 0.998 | 0.96 | 0.94, 0.99 | 0.007 |  |
| **Sex – Age Interaction** |  |  |  |  |  |  |  |  |  |  |
| Female per Year |  |  |  | 0.92 | 0.84, 1.02 | 0.12 |  |  |  |  |
| **Parents ADHD** |  |  |  |  |  |  |  |  |  |  |
| No | Reference | . | . |  |  |  | Reference | . | . |  |
| Yes | 2.07 | 1.41, 3.04 | <0.001 |  |  |  | 1.6 | 1.2, 2.12 | 0.001 |  |
| **Mother Metabolic** |  |  |  |  |  |  |  |  |  |  |
| No Diabetes | Reference | . | . |  |  |  | Reference | . | . |  |
| Diabetes | 1.27 | 0.89, 1.80 | 0.19 |  |  |  | 1.19 | 0.93, 1.52 | 0.16 |  |
| **Parents Maximum Education** |  |  |  |  |  |  |  |  |  |  |
| Graduate |  |  |  | Reference | . | . |  |  |  |  |
| Bachelor |  |  |  | 0.80 | 0.49, 1.31 | 0.37 |  |  |  |  |
| Some college |  |  |  | 1.27 | 0.81, 2.01 | 0.30 |  |  |  |  |
| High School or Less |  |  |  | 1.59 | 0.81, 3.13 | 0.18 |  |  |  |  |
